# Skeletal Muscle Mass Is Independently Associated With Water Turnover in Male Athletes

**DOI:** 10.1111/sms.70332

**Published:** 2026-06-30

**Authors:** Hiroyuki Sagayama, Yoko Tanabe, Akiko Uchizawa, Suraiya Khatun, Keisuke Shiose, Rie Tomiga‐Takae, Jun Yasukata, Yasuki Higaki, Takahiro Ohnishi, Hideyuki Takahashi, Yosuke Yamada, Analiza M. Silva, Emi Kondo

**Affiliations:** ^1^ Institute of Health and Sport Sciences, University of Tsukuba Ibaraki Japan; ^2^ Advanced Research Initiative for Human High Performance (ARIHHP) University of Tsukuba Ibaraki Japan; ^3^ Department of Life Sciences The University of Tokyo Tokyo Japan; ^4^ Japan Institute of Sports Sciences Tokyo Japan; ^5^ Japan Society for the Promotion of Science Tokyo Japan; ^6^ Faculty of Education University of Miyazaki Miyazaki Japan; ^7^ Center for General Education Kagoshima University Kagoshima Japan; ^8^ Faculty of Sports and Health Science Fukuoka University Fukuoka Japan; ^9^ Graduate School of Medicine Tohoku University Sendai Miyagi Japan; ^10^ Exercise and Health Laboratory, CIPER, Faculdade de Motricidade Humana Universidade de Lisboa Cruz‐Quebrada Portugal; ^11^ Department of Health and Sport Sciences Osaka University of Health and Sport Sciences Osaka Japan

**Keywords:** athletes, body composition, intracellular water, regularized regression, skeletal muscle mass, stable isotope technique, water turnover

## Abstract

Fat‐free mass (FFM) is a key component of body composition that contributes moderately to water turnover (WT). However, FFM consists of multiple components, and the extent of its contribution to WT, especially in relation to skeletal muscle mass (SMM) in athletes, remains unclear. This study aimed to examine the association between WT and body composition in athletes. Thirty male participants (20 ± 2 years old) were members of college sports clubs or athletic organizations. WT was determined using the deuterium dilution method for 1 week. Total body water (TBW), intracellular water (ICW), and extracellular water (ECW) were measured using deuterium and sodium bromide dilution. Bone mineral content was measured using DXA, and four‐component models were used to calculate FFM and total body protein (TBP). MRI and MRS were used to evaluate SMM and muscle glycogen. WT was positively associated with TBW (*r* = 0.563), ICW (*r* = 0.549), SMM (*r* = 0.548), and ambient temperature (*r* = 0.519). To overcome mathematical coupling and multicollinearity, regularized regression analyses (LASSO and Elastic Net) were performed. Because TBW and TBP are algebraically dependent on the deuterium‐derived water space from which WT was calculated, they were excluded from the candidate predictors. Both models retained SMM, ICW, and ambient temperature as predictors of WT, whereas ECW, bone mineral content, muscle glycogen, and relative humidity were not retained. The modest cross‐validated performance (leave‐one‐out *R* ^2^ = 0.20–0.23) suggests that WT in athletes is also influenced by behavioral and unmeasured environmental factors. Functional body composition compartments, specifically SMM and ICW, were associated with WT in athletes beyond the mathematical dependency between WT and TBW; SMM, measured independently by MRI, was an independent correlate, whereas the association of ICW—derived in part from TBW—was not fully independent of the WT measurement.

## 1 Introduction

Body water turnover (WT) is a dynamic physiological indicator reflecting the continuous exchange of water between the body and its environment, essential for sustaining metabolic homeostasis [1]. Large‐scale studies using international databases of the doubly labeled water (DLW) method have shown globally that WT is determined by multiple factors, including age, physical activity, and climate conditions such as temperature, humidity, and altitude [2]. In older adults, factors such as fat‐free mass (FFM), total energy expenditure (TEE), and physical activity levels have also been confirmed as significant predictors of WT [3]. Across diverse populations, FFM has consistently been highlighted as the largest internal determinant of WT, reflecting its role as the principal reservoir of body water and the primary site of metabolically active tissue [2, 4].

In athletes and highly active populations, however, the physiological determinants of WT extend well beyond those observed in the general population. Rigorous training imposes substantial demands on thermoregulatory, renal, and metabolic systems, fundamentally altering the dynamics of water flux [5]. During exercise, skeletal muscle generates large amounts of heat, triggering pronounced sweating responses that represent the primary route of water loss. In parallel, trained athletes exhibit enhanced sweat gland sensitivity and increased sweat rates relative to untrained individuals, reflecting chronic thermoregulatory adaptations to repeated heat stress [5]. Fluid intake behavior is similarly modified; athletes adopt deliberate hydration strategies before, during, and after exercise to compensate for sweat‐induced losses and support performance [6, 7]. Furthermore, renal water conservation mechanisms are activated under exercise‐induced dehydration, with antidiuretic hormone promoting free water reabsorption and minimizing urinary losses [6]. The integrated action of these mechanisms—increased sweat output, heightened fluid intake, and renal conservation—collectively drives WT to substantially higher levels in athletes compared with sedentary populations.

DLW studies on extreme ultramarathons [8], continuous ultra‐endurance races in cold weather [9], high‐altitude mountaineering [10], severe cold‐regions races [11], and various competitive athletes [12, 13] have confirmed that extreme TEE and the accompanying heat production elevate WT to exceptional levels [14]. These findings underscore the extent to which training load and energy expenditure amplify water requirements in athletic populations.

A key methodological consideration in understanding the determinants of WT concerns the structural relationship between WT and total body water (TBW). WT is calculated as the product of the deuterium dilution space (Nd) and the elimination rate (kd) (WT = Nd × kd) [15, 16], FFM is subsequently derived from TBW by assuming a constant hydration fraction of approximately 73% [17, 18]. This means that associations between WT and TBW or FFM inherently reflect a degree of mathematical coupling arising from shared terms in their calculation. Importantly, this structural dependency does not diminish the physiological relevance of TBW as the principal body water reservoir underlying water flux; rather, it highlights the need to identify which specific body composition compartments best explain the variability in WT beyond this dependency.

In athletes, skeletal muscle mass (SMM) is considered a particularly compelling candidate as a functional determinant of dynamic WT. Skeletal muscle is the primary heat‐producing organ during exercise, directly triggering water loss through sweating [19, 20]. In addition, muscle glycogen is stored together with a large amount of intracellular water (ICW) [21]. ICW is a molecular body composition compartment that reflects the water contained within cells and has emerged as an indicator of metabolically active cell mass, predominantly skeletal muscle, in contemporary body composition research. Given that SMM supports high TEE and thermoregulatory demands, we hypothesized that SMM, measured independently of the TBW estimation process, is an independent determinant of WT variability in athletes and that ICW, as the intracellular compartment of metabolically active tissue, is also associated with WT, contributing explanatory power beyond TBW alone.

To test this hypothesis, the present study employs magnetic resonance imaging (MRI), a criterion method for body composition, to directly quantify SMM as an independent variable that does not rely on the TBW estimation process. By statistically accounting for the structural dependency between WT and TBW, we separately examine the contributions of SMM and ICW to WT variability. Therefore, the purpose of this study is to clarify how these functional body composition compartments, particularly SMM measured independently of TBW, explain the variability of dynamic WT of athletes.

## 2 Materials and Methods

### 2.1 Study Design and Participants

This cross‐sectional study was conducted at the Japan Institute of Sports Sciences (Tokyo, Japan). A total of 30 healthy, active young male adults were enrolled. The participants were athletes or active individuals engaged in regular physical training (e.g., cycling, triathlon, wrestling, and judo). Participants were included if they were not using any medications or supplements, had no history of thyroid or cardiac diseases, and had no extreme dietary restrictions. Thirty participants were included in the final analyses (Table 1). The participants were invited to attend an informational meeting where they were informed of the benefits and potential risks of the investigation. All participants provided written informed consent. The study protocol was approved by the Institutional Review Board of the Japan Institute of Sports Sciences (Approval No.: 050). This study was performed in accordance with the ethical guidelines of the Declaration of Helsinki.

**TABLE 1:** Characteristics, body composition, and environmental conditions.

Variable	Mean ± SD
*Physical characteristics*	
Age, years	20 **±** 2
Height, cm	170.7 **±** 6.0
Body mass, kg	68.9 **±** 10.0
Body volume, L	64.4 **±** 9.8
Body density, g/cm^3^	1.0693 **±** 0.0104
*Body composition*	
FFM, kg	59.8 **±** 7.0
FM, kg	9.1 **±** 4.1
TBW, kg	43.5 **±** 4.8
ICW, kg	24.2 **±** 3.2
ECW, kg	19.4 **±** 2.7
SMM, kg	32.5 **±** 3.9
TBP, kg	13.5 **±** 2.0
BMC, kg	2.7 **±** 0.4
Muscle glycogen, mM	89.1 **±** 23.3
*Water & environment*	
WT, L/day	4.48 ± 1.38
Ambient temperature, °C	15.9 **±** 8.4
Relative humidity, %	69.8 **±** 13.3

*Note:* Values are mean ± SD. WT and body water variables were determined by ^2^H and NaBr dilution. SMM was measured by whole‐body MRI. FFM was calculated using a 4‐component model. Muscle glycogen was assessed by ^13^C‐MRS; *n* = 25 for muscle glycogen.

Abbreviations: BMC, bone mineral content; ECW, extracellular water; FFM, fat‐free mass; FM, fat mass; ICW, intracellular water; SMM, skeletal muscle mass; TBP, total body protein; TBW, total body water; WT, water turnover.

### 2.2 Measurement of SMM by MRI

Whole‐body SMM was determined using MRI. Continuous transverse images were obtained from the humerus to the ankle using a 3.0‐T scanner (MAGNETOM Skyra; Siemens Healthineers, Erlangen, Germany). The cross‐sectional areas of the skeletal muscle were segmented manually and semi‐automatically using image analysis software (Attractive Basic 3D; PixSpace Ltd., Tokyo, Japan), incorporating threshold, region growing, mathematical morphology, and snakes functions. The total muscle volume was calculated. SMM (kg) was calculated by multiplying the muscle volume by the density of fresh skeletal muscle (1.041 g/cm^3^). The whole‐body MRI protocol used in the present study has been applied and validated against independent reference methods in our previous work, showing close agreement with DXA‐based estimation equations and the D3‐creatine dilution method [22, 23]. Regarding measurement error, the reproducibility of whole‐body MRI muscle quantification has been reported to be excellent: test–retest intraclass correlation coefficients approach 1.0, with 95% limits of agreement within approximately 1.8%–6.6% of the mean for automated measurement [24], and the reproducibility of MRI and CT estimates of adipose tissue‐free skeletal muscle has been reported to be approximately 2% [25]. As our SMM was obtained using a semi‐automated approach, the expected measurement error is considered to fall within this range.

### 2.3 Measurement of WT and Body Water Compartments

TBW and WT were determined using the deuterium (^2^H) dilution technique. Each participant consumed a weighed dose of ^2^H_2_O (0.12 g/kg estimated TBW). Urine samples were collected over an observation period to determine the ^2^H dilution space (Nd) and its elimination rate (kd). TBW was calculated from Nd with correction for in vivo isotope exchange, with a reported coefficient of variation (CV) of 0.6% [26]. WT (L/day) was calculated as the product of Nd and kd (WT = Nd × kd), corrected for fractionation effects [27, 28]. ECW was determined using the sodium bromide (NaBr) dilution method. ICW was calculated as the difference between TBW and ECW (ICW = TBW—ECW) [29]. During the observation period, ambient temperature and relative humidity were obtained from the Japan Meteorological Agency database (JMA; https://www.data.jma.go.jp/risk/obsdl/index.php) using the meteorological observation station nearest to each participant's residential area. No formal assessment of hydration status was performed during the observation period; however, participants were instructed to maintain their habitual dietary and fluid intake throughout.

### 2.4 Measurement of Body Composition, Total Body Protein, and Muscle Glycogen

Bone mineral content (BMC) was determined via whole‐body dual‐energy X‐ray absorptiometry (DXA; QDR 4500, Discovery A, fan‐beam scanner, software version 12.7.3.2; Hologic, MA, USA) [22] with a reported CV of 0.5%. Body volume was assessed using air‐displacement plethysmography (BOD POD, COSMED, Italy) with a reported CV of 0.1% [30]. Total body protein (TBP) was estimated using a four‐component (4C) model, integrating measured values of TBW, body density, and BMC [18]. The propagation of measurement error for the 4C model was estimated following the method described by Santos et al. [31]. Based on the CVs for TBW (0.6%), body volume (0.1%), and BMC (0.5%) from our laboratory, and the mean values observed in this sample (TBW = 43.5 kg, body volume = 64.4 L, BMC = 2.7 kg), the estimated standard deviation in fat mass derived from the 4C model was 0.25 kg (CV = 2.7%), and that for TBP was 0.36 kg (CV = 2.7%). Additionally, muscle glycogen concentration in the thigh was measured using ^13^C‐magnetic resonance spectroscopy (^13^C‐MRS) with a 3‐T MR system (Magnetom Verio, Siemens, Germany) as previously described [32], with a reported CV of 3.5% in our laboratory.

### 2.5 Statistical Analysis

Results are presented as mean ± standard deviation (SD). The normality of each variable was assessed using the Shapiro–Wilk test. Because some variables, including WT, deviated from normality, both Pearson's correlation coefficient (*r*) and Spearman's rank correlation coefficient (rho) were calculated to evaluate the relationships between WT and physical/environmental parameters, and both are presented in Table 2. To identify the primary determinants of WT and address potential multicollinearity among the body composition variables, regularized regression analyses—specifically Least Absolute Shrinkage and Selection Operator (LASSO) and Elastic Net regression—were performed [33, 34]. These regularization methods are useful for selecting relevant variables and improving model stability, especially when the predictors are highly correlated and the sample size is small. The candidate predictor variables included SMM, ICW, ECW, BMC, muscle glycogen, ambient temperature, and relative humidity, while WT was used as the dependent variable. TBW and TBP were excluded from the candidate predictors because they are algebraically dependent on the deuterium‐derived water space from which WT was measured, which would confound the interpretation of variable selection. FFM and body mass were also excluded because they encompass SMM and the other compartments, respectively. Missing muscle glycogen values (*n* = 5) were imputed by multiple imputation using predictive mean matching. All predictors were standardized (mean 0, unit variance) within the algorithm before penalization, and coefficients are reported on the original scale. A 10‐fold cross‐validation method was applied to determine the optimal penalty parameter (*λ*) with the lowest prediction error (lambda.min). Separate LASSO (*α* = 1) and Elastic Net (*α* = 0.5) models were developed using the glmnet package, and variables retaining non‐zero coefficients at the optimal *λ* value were considered retained predictors of WT. Model performance was evaluated by leave‐one‐out cross‐validation (cross‐validated *R* ^2^ and root mean square error), with in‐sample values reported for comparison. Selection stability was assessed using 1000 bootstrap resamples, and the selection frequency of each predictor was recorded. All statistical analyses and figure generation were conducted using R software (version 4.6.0). Statistical significance for basic tests was set at *p* < 0.05.

**TABLE 2:** Pearson and Spearman correlation coefficients for water turnover.

Variables	Pearson *r*	*p*	Spearman *ρ*	*p*
*Body composition*				
SMM, kg	0.548	0.002	0.492	0.006
FFM, kg	0.566	0.001	0.389	0.033
TBP, kg	0.546	0.002	0.327	0.077
ICW, kg	0.549	0.002	0.529	0.003
ECW, kg	0.348	0.059	0.230	0.222
TBW, kg	0.563	0.001	0.422	0.020
Muscle glycogen, mM	0.110	0.602	0.337	0.100
*Environment*				
Ambient temperature, °C	0.519	0.003	0.361	0.050
Relative humidity, %	0.312	0.094	0.230	0.221

Abbreviations: ECW, extracellular water; FFM, fat‐free mass; ICW, intracellular water; SMM, skeletal muscle mass; TBP, total body protein; TBW, total body water.

## 3 Results

### 3.1 Participant Characteristics and Environmental Conditions

The physical characteristics of the participants and the environmental conditions during the study period are summarized in Table 1. The mean age, height, and body mass were 20 ± 2 years, 170.7 **±** 6.0 cm, and 68.9 **±** 10.0 kg, respectively. SMM measured by MRI was 32.5 **±** 3.9 kg. The average WT was 4.48 **±** 1.38 L/day (range: 2.82–7.60 L/day). The ambient temperature and relative humidity during the observation period were 15.9°C **±** 8.4°C (range: 5.1°C–28.8°C) and 69.8% **±** 13.3% (range: 44.6%–85.1%), respectively.

### 3.2 Correlation Between WT and Physical/Environmental Parameters

**FIGURE 1:**
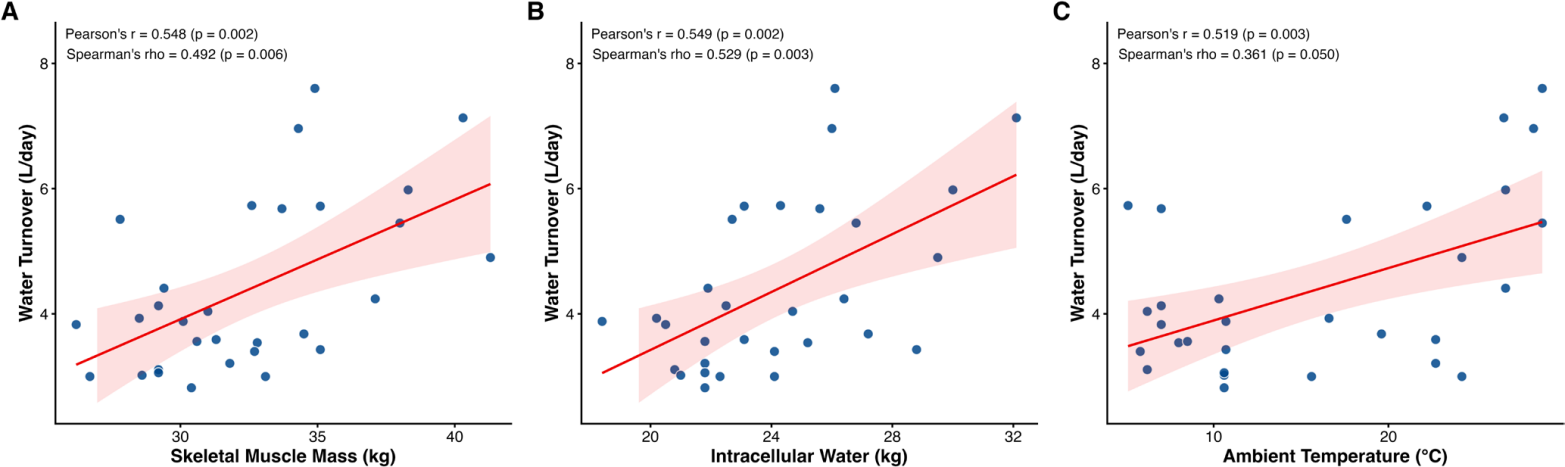
Positive associations of water turnover with skeletal muscle mass, intracellular water, and ambient temperature. Scatter plots illustrate the positive correlations between daily water turnover and (A) skeletal muscle mass, (B) intracellular water, and (C) ambient temperature. Solid red lines represent the linear regression lines of best fit, and the shaded areas indicate the 95% confidence intervals. Pearson's correlation coefficients (*r*) and Spearman's rank correlation coefficients (rho), along with their corresponding *p*‐values, are displayed for each variable.

The relationships between WT and various predictors are shown in Table 2 and Figure 1. WT was significantly and positively correlated with SMM (*r* = 0.548, *p* < 0.01; Figure 1A) and ICW (*r* = 0.549, *p* < 0.01; Figure 1B). Ambient temperature also showed a significant positive correlation with WT (*r* = 0.519, *p* < 0.01; Figure 1C). Although TBW was strongly correlated with WT (*r* = 0.563, *p* < 0.01), no significant correlations were observed between WT and ECW, muscle glycogen, or relative humidity.

### 3.3 Variable Selection for WT Using Regularized Regression

To identify the most robust predictors of WT, LASSO and Elastic Net regression analyses were performed (Table 3). TBW and TBP were excluded from the candidate predictors because of their algebraic dependence on the deuterium‐derived water space from which WT was measured. Both models consistently selected SMM, ICW, and ambient temperature as retained predictors, while ECW, BMC, muscle glycogen, and relative humidity were excluded (*β* = 0). All three retained predictors showed positive coefficients in both models. As predictors were measured on different scales, the magnitude of the unstandardized coefficients should not be directly compared across variables to infer relative importance. In the bootstrap analysis, ambient temperature and ICW were the most stable predictors (selection frequencies of 81%–85% and 78%–89%, respectively), followed by SMM (56%–73%), whereas the non‐retained variables were each selected in less than 50% of resamples. Cross‐validated performance was modest (leave‐one‐out *R* ^2^ = 0.20 and 0.23 for LASSO and Elastic Net, respectively), and was lower than the in‐sample *R* ^2^ (0.39 for both models).

**TABLE 3:** Variable selection for water turnover prediction using LASSO and Elastic Net regularized regression models.

Predictor	LASSO coefficient	Elastic Net coefficient	Selection frequency, LASSO (%)	Selection frequency, Elastic Net (%)	Selected by both
Intercept	−0.005	−0.012	—	—	—
SMM, kg	0.034	0.047	55.5	72.7	Yes
ICW, kg	0.111	0.096	77.9	88.7	Yes
Temp, °C	0.044	0.042	81.2	84.9	Yes
ECW, kg	0	0	39.2	46.7	No
BMC, kg	0	0	34.4	40.9	No
Glycogen, mM	0	0	43.9	49.2	No
Humidity, %	0	0	34.9	41.7	No
Model statistics					
*R* ^2^, LOOCV	0.202	0.225			
RMSE, LOOCV	1.214	1.197			
*R* ^2^, in‐sample	0.392	0.389			
RMSE, in‐sample	1.059	1.062			

*Note:* Coefficients are reported on the original scale. Selection frequency is the percentage of 1000 bootstrap resamples in which each predictor was retained. ‘Selected by both’ indicates non‐zero coefficients in both models.

Abbreviations: BMC, bone mineral content; ECW, extracellular water; Elastic Net, elastic net regularized regression; ICW, intracellular water; LASSO, least absolute shrinkage and selection operator; LOOCV, leave‐one‐out cross‐validation; RMSE, root mean square error; SMM, skeletal muscle mass; Temp, ambient temperature.

## 4 Discussion

WT is a crucial indicator of water dynamics that support life and exercise performance. While previous large‐scale research has shown globally [2] that WT is determined by age, body size, physical activity, and environmental factors, this study focused specifically on athletes and examined its internal determinants in detail using regularized regression models. The regularized models retained SMM, measured independently by MRI, together with ICW and ambient temperature as predictors of WT. These findings suggest that WT in athletes may not simply depend on the size of the body as a container but may be partly associated with skeletal muscle, a metabolically active functional tissue.

In previous studies using the DLW method, FFM was considered the largest determinant of WT. However, as Westerterp [15] and Wang et al. [17] pointed out, FFM derived from the isotope dilution method is based on the fixed assumption that about 73% of FFM consists of water. Since WT is also calculated from the deuterium dilution space, associations observed in prior studies may have been influenced by structural mathematical coupling between these variables. In this study, SMM was quantified directly by MRI and is therefore fully independent of the deuterium‐derived water space used to calculate WT. To avoid this coupling, TBW and TBP—both algebraically dependent on that water space—were excluded from the candidate predictors. SMM was retained as a predictor of WT, indicating that it carries explanatory information for WT that is not attributable to the mathematical dependency inherent in TBW‐based estimates. Among the retained predictors, SMM is the only body composition variable that is entirely free of this coupling.

In the simple correlation analysis, TBW showed a strong positive correlation with WT (*r* = 0.563). However, this largely reflects a mathematical dependency because the WT calculation is based on the deuterium dilution space, which is also used to derive TBW. For this reason, TBW was not entered as a candidate predictor, as it corresponds almost entirely to that water space and would produce a near‐complete coupling with WT. ICW, calculated as TBW minus ECW, shares a partial dependency with the WT measurement. We acknowledge this limitation; however, unlike TBW, ICW distinguishes the intracellular fraction associated with metabolically active tissue from total body water, and its retention in preference to TBW and ECW suggests that the intracellular compartment, rather than total or extracellular water, is more closely associated with WT.

The selection of SMM and ambient temperature as retained predictors of WT is physiologically reasonable. Skeletal muscle is the primary organ for metabolic heat production during physical activity [20]. Furthermore, previous studies have shown that athletes who train daily have remarkably developed peripheral sweat gland functions compared to untrained individuals, resulting in an enhanced sweating response (cooling function) to heat stress [19, 35]. In addition to this output‐driven mechanism, SMM may contribute to WT from the input side through the production of metabolic water. Skeletal muscle is highly abundant in mitochondria [36, 37], and athletes with high physical activity oxidize substantial amounts of substrates to meet extreme energy demands, which could generate endogenous metabolic water during oxidative phosphorylation. Recent studies in extreme cold environments have also demonstrated that high TEE drives high WT regardless of ambient temperature [9]. Since WT measured by the deuterium dilution encompasses total water flux, this internal generation of metabolic water may contribute to baseline WT [2, 38]. Because TEE, metabolic heat production, and metabolic water generation were not measured in the present study, these mechanisms are proposed as hypotheses rather than demonstrated explanations.

In addition to external environmental factors, the retention of ICW complements the functional role of SMM. Most ICW is held within skeletal muscle cells, and when muscles store glycogen, about 3–4 g of water are drawn in for every 1 g of glycogen [39]. In this study, muscle glycogen content was directly measured using ^13^C‐MRS, but it was not retained as a predictor of WT. There are two possible reasons. First, there is a discrepancy in the time axis: the MRS measurement represents a static storage amount at one point in time, whereas WT measured by the deuterium dilution reflects the dynamic flow of water over about a week. Muscle glycogen fluctuates greatly day to day due to intense training and nutrient intake, and a snapshot value at the time of measurement may not necessarily represent the average weekly water turnover. Second, MRS is a local indicator evaluating only specific muscles, whereas ICW comprehensively reflects water dynamics in all muscles of the body. Statistically, ICW may already incorporate the water associated with glycogen storage, so that ICW—a more stable, whole‐body indicator—was retained in preference to local glycogen content.

On the other hand, the variance in WT explained by the regularized models in this study was modest (cross‐validated *R* ^2^ = 0.20 and 0.23 for the LASSO and Elastic Net models, respectively), substantially lower than the in‐sample *R* ^2^ of 0.39, indicating that the apparent fit was partly optimistic given the small sample size, and there are still large individual differences (residuals) in the WT of athletes [8, 12]. This unexplained variance is likely due to the highly dynamic behavioral and environmental factors of water balance specific to athletes. Researchers have pointed out that there are significant individual differences in the perception of thirst and voluntary water ingestion immediately after intense interval training or continuous exercise [40, 41]. Case studies of 161‐km ultramarathons have shown that even with extreme WT, athletes often experience negative fluid balance due to the limits of ingestion [8]. The moderate explained variance suggests that additional factors not captured in the present models contribute to WT variability in athletes. Individual differences in hydration status—which were not formally assessed in this study—may explain part of the remaining variability. As Armstrong and Johnson [42] highlighted, chronic differences in habitual fluid intake and renal water conservation capacity may independently influence WT estimates derived from the isotope method. Therefore, while SMM may be related to potential water metabolic capacity, the final daily WT should be interpreted as being determined by the complex interaction between these functional body compartments, behavioral and environmental factors.

Several limitations should be acknowledged. First, the cross‐sectional design precludes causal inference; the observed associations between SMM, ICW, and WT do not establish that changes in these compartments prospectively drive changes in WT. Longitudinal studies are needed to examine this question. Second, ICW was derived as TBW minus ECW and therefore retains a partial dependency on the deuterium‐derived water space from which WT was calculated; its association with WT should be interpreted with this caveat, whereas SMM, measured by MRI, is free of this dependency. Third, no formal assessment of hydration status was performed during the observation period, and individual differences in habitual fluid intake or chronic hydration status may have contributed to the unexplained variability in WT. Fourth, the present study was limited to young male athletes, and the findings may not be generalizable to female athletes or other populations. Fifth, the relatively small sample size (*n* = 30) may have limited statistical power, despite the use of cross‐validation in the regularized regression models. Sixth, WT was measured using deuterium dilution rather than the DLW method; because the rate of CO_2_ production was not available, metabolic water and respiratory and transcutaneous water losses could not be estimated, and preformed water intake could not be partitioned from total WT [12, 13]. Seventh, laboratory‐specific test–retest reproducibility of MRI‐derived SMM was not assessed, although the protocol has been validated against independent reference methods in our previous work, and the reported reproducibility of whole‐body MRI muscle quantification is high.

## 5 Perspective

The present findings contribute to evidence suggesting that body composition beyond TBW is relevant to water metabolism in athletes. While large‐scale DLW studies have consistently identified FFM as the primary determinant of WT in the general population [2], the present study extends this understanding by demonstrating that SMM—assessed by MRI independently of the deuterium‐derived water space—is independently associated with WT in athletes after accounting for mathematical coupling, while ICW, a compartment reflecting the water held within metabolically active cells, showed a related but not fully independent association. These findings align with recent DLW studies demonstrating exceptionally high WT in extreme endurance athletes [8, 9] and suggest that the metabolic and thermoregulatory demands of skeletal muscle may be related to water requirements beyond what is predicted by total body water volume alone. Future longitudinal studies examining training‐induced changes in SMM and ICW and their relationship to WT are warranted. If confirmed, SMM assessment could serve as a practical indicator of water requirements in athletic populations, with potential applications in individualized hydration management.

## Author Contributions

H.S., A.U., S.K., A.M.S., K.S., R.T.‐T., and E.K. planned the study, provided significant design ideas, and acquired funding. H.S., Y.T., T.O., H.T., and E.K. collected the data. R.T.‐T., J.Y., and Y.H. performed and organized the stable isotope specimen analyses and provided IRMS resources. T.O. and H.T. analyzed the magnetic resonance data and operated the systems. H.S., A.U., S.K., and Y.Y. conducted the statistical analyses. H.S., A.U., and S.K. prepared the illustrations and drafted the manuscript. All authors interpreted the results and revised and approved the final version of the manuscript.

## Funding

This investigation was mainly supported by the JSPS KAKENHI (16J11877, 18K17882 and 23KK0177 to HS). This work was also supported by the Advanced Research Initiative for Human High Performance (ARIHHP), University of Tsukuba.

## Conflicts of Interest

The authors declare no conflicts of interest.

## Data Availability

The data that support the findings of this study are available on request from the corresponding author. The data are not publicly available due to privacy or ethical restrictions.
